# Calibration Alignment Sensitivity in Corneal Terahertz Imaging

**DOI:** 10.3390/s22093237

**Published:** 2022-04-22

**Authors:** Faezeh Zarrinkhat, Mariangela Baggio, Joel Lamberg, Aleksi Tamminen, Irina Nefedova, Juha Ala-Laurinaho, Elsayed E. M. Khaled, Juan M. Rius, Jordi Romeu, Zachary Taylor

**Affiliations:** 1 CommSensLab, Technical University of Catalonia/UPC, 08034 Barcelona, Spain; juan-manuel.rius@upc.edu (J.M.R.); jordi.romeu-robert@upc.edu (J.R.); 2 Department of Electronics and Nanoengineering, Millilab, Aalto University, 02150 Espoo, Finland; mariangela.baggio@aalto.fi (M.B.); joel.lamberg@aalto.fi (J.L.); aleksi.tamminen@aalto.fi (A.T.); irina.nefedova@aalto.fi (I.N.); juha.ala-laurinaho@aalto.fi (J.A.-L.); zachary.taylor@aalto.fi (Z.T.); 3 Department of Electrical Engineering, Assiut University, Assiut 71515, Egypt; esamk54_2000@aun.edu.eg; 4 High Institute of Engineering and Technology, Sohag 82524, Egypt

**Keywords:** cornea, particle-swarm optimization, terahertz

## Abstract

Improving the longitudinal modes coupling in layered spherical structure contributes significantly to corneal terahertz sensing, which plays a crucial role in the early diagnosis of cornea dystrophies. Using a steel sphere to calibrate reflection from the cornea sample assists in enhancing the resolution of longitudinal modes. The requirement and challenges toward applying the calibration sphere are introduced and addressed. Six corneas with different properties are spotted to study the effect of perturbations in the calibration sphere in a frequency range from 100 GHz to 600 GHz. A particle-swarm optimization algorithm is employed to quantify corneal characteristics considering cases of accurately calibrated and perturbed calibrated scenarios. For the first case, the study is carried out with signal-to-noise values of 40 dB, 50 dB and 60 dB at waveguide bands WR-5.1, WR-3.4, and WR-2.2. As expected, better estimation is achieved in high-SNR cases. Furthermore, the lower waveguide band is revealed as the most proper band for the assessment of corneal features. For perturbed cases, the analysis is continued for the noise level of 60 dB in the three waveguide bands. Consequently, the error in the estimation of corneal properties rises significantly (around 30%).

## 1. Introduction

There has been a surge in THz application in medical imaging and sensing. One of these promising areas is using this spectrum for quantification of the human cornea water content and thickness, which contributes to the early diagnosis of cornea dystrophies [1,2,3,4,5,6]. The nominal cornea dimension (0.5 mm corneal phantom sitting on a 7.5 mm aqueous core) manifests as a lossy thin film at THz frequencies, hence the extraction of corneal central thickness (CCT), corneal anterior water content (CAWC), and corneal posterior water content (CPWC) is achievable through the coupling of longitudinal modes via broadband reflectometry [7]. Given the cornea anatomy acting as a half-space structure in THz band, back-scattered field from the cornea is achievable by stratified medium theory [8,9,10], although some challenges such as phase matching in peripheral areas needed to be addressed. A study based on Fourier optics and vector spherical harmonics enables accurate calculation of back-scattered field from the cornea, by modelling cornea as a layered sphere under Gaussian illumination [11,12]. In practice, the two-way propagation through the reflectometer quasioptics is frequency-dependent, and calibrator targets with known reflectivity are necessary. The studies suggest using a PEC sphere identical to the cornea radius of curvature (ROC) and center of curvature (COC) for calibration [9,12], but reaching this identical situation comes with challenges.

Extracting cornea properties from the back-reflected beam (computed by stratified medium theory) is proposed using a particle-swarm optimization (PSO) algorithm and suggests the WR-5.1 frequency band is more compatible for corneal sensing in [13,14].

In this paper, the theory to compute the coupling efficiency as criteria of resolution of longitudinal modes is briefly described. Six corneal properties which are targeted for study are introduced and compared with the plane-wave model after correct calibration. Different scenarios that can perturb the calibration with the PEC sphere in a realistic situation are introduced and the consequences of these perturbations are addressed by comparing with the plane-wave model. Next, the PSO algorithm is applied to correctly calibrated simulation with added signal to noise ratios (SNRs), to quantify target properties in different waveguide bands. Finally, the root-mean-square deviation (RMSD) of six targets parameters after perturbed calibration in different waveguide bands are reported in the tables.

## 2. Theory Based on Fourier Optic and Vector Spherical Harmonics

To solve the scattering problem of a layered sphere (representing a cornea) under Gaussian illumination, the methodology introduced in [15,16,17] appears to be the best choice since the corneal dimensions are of the order of the wavelength of the incident beam. The incident field $E^{i}$ and scattered field $E^{s}$ are obtained as:

(1) $$E^{i}=\sum_{m}\sum_{n}D\left[a^{e}M_{e}^{1}+a^{o}M_{o}^{1}+b^{e}N_{e}^{1}+b^{o}N_{o}^{1}\right],$$

(2) $$E^{s}=\sum_{m}\sum_{n}D\left[f^{e}M_{e}^{3}+f^{o}M_{o}^{3}+g^{e}N_{e}^{3}+g^{o}N_{o}^{3}\right].$$

where $a^{e}$, $a^{o}$, $b^{e}$, and $b^{o}$ are the incident field coefficients. The $f^{e}$, $f^{o}$, $g^{e}$, and $g^{o}$ are scattered field coefficients. The *D* is a normalization factor. The *n* and *m* represent radial and azimuthal mode numbers. The $M^{1}$, $N^{1}$, $M^{3}$, and $N^{3}$ are vector spherical harmonics of first and third kind [18]. A through details of the theory can be found in [11,12,15,16,17,19].

Coupling between the incident and back-scattered field (along optical axis *z*) are calculated by:

(3) $$CE=\frac{\int \int E^{i}.E^{s}dxdy}{\int \int E^{i}.E^{i*}dxdy},$$

in a plane perpendicular to the optical axis, where * denotes the complex conjugate and the incident field $E^{i}$ and scattered field $E^{s}$ described by Equations (1) and (2).

## 3. Cornea Calibration

### 3.1. Correctly Calibrated Cornea

**Figure 1:**
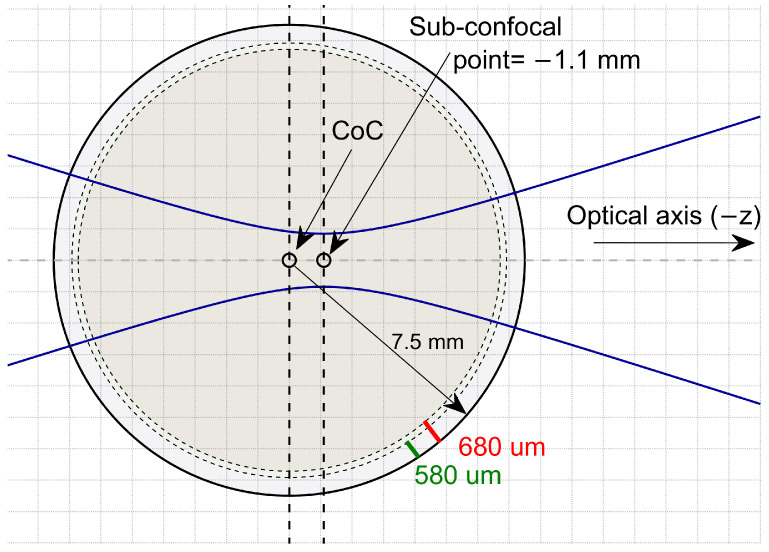
The cornea is illuminated by a Gaussian beam in a way that the beam focus on the sub-confocal point. The cornea radius is 7.5 mm. Corneal phantom thickness is either 580 μm or 680 μm discretized to 29 and 34 equally distanced 20 μm layers, respectively.

Cornea modelled as a layered corneal phantom sitting on an aqueous core with different characteristics listed in Table 1. The cornea ROC is set as 7.5 mm at the anterior and the cornea COC is fixed on $x_{0}=0$, $y_{0}=0$, and $z_{0}=0$. Corneal phantom thickness is considered either 580 μm or 680 μm corresponding to posterior radius of 6.92 μm and 6.82 μm, as shown in Figure 1. Aqueous-humour permittivity is derived from the double-Debye model [20].

For the corneal phantom, the gelatin hydrogel characterized in [21] is used, where the complex permittivity is a function of water content. The permittivity model is based on the effective medium theory and constitutes two main components: free water and solid content; the latter is a combination of dry gelatin and bound water. Furthermore, it is assumed that the total water content varies linearly from the drier anterior surface to the more hydrated posterior surface. The cornea is discretized at most 20 μm-thick layers, whose permittivity depends on the linear water gradient. Hence, for the phantom thickness of 580 μm, cornea modeled as a core with 29 number of equally distanced layers and for the thickness of 680 μm, cornea modelled as a core with 34 number of equally distanced layer.

**Figure 2:**
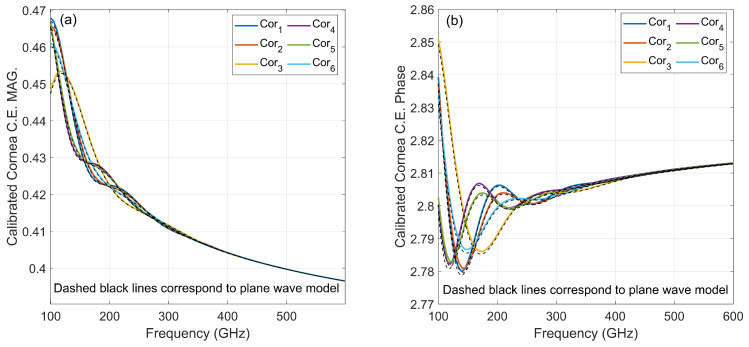
Correctly calibrated cornea coupling efficiency (**a**) magnitude and (**b**) phase are compared with stratified medium theory (shown in black-dashed line) at frequency range of 100–600 GHz.

Each cornea coupling coefficient is computed with Equation (3) and calibrated against the coupling coefficient of the same size steel sphere, which is accurately located in the cornea location (called Cal${}_{0}$), for the frequency range from 100 GHz to 600 GHz. Targets are illuminated by a Gaussian beam in a way that the beam focus on the sub-confocal point introduced in [10,12], shown in Figure 1. Figure 2 compares calibrated efficiency with the stratified medium theory which models the cornea as a planar half-space illuminated by plane wave [8] at the frequency range of 100–600 GHz. Due to the proper matching between plane-wave phase front and planar boundary, the plane-wave calculation is considered as a reference offering the maximum coupling between the incident and back-scattered field.

Figure 2 shows two important points. First, correct calibration (calibration sphere has identical ROC and COC to the target) delivers a very close coupling coefficient to reference plane-wave model coupling, leading to a high resolution of longitudinal modes. Second, the difference between each target is more distinguishable in lower frequencies.

### 3.2. Perturbed Calibrated Cornea

As mentioned in the previous subsection, an accurate calibration leads to reaching a resolution close to maximum coupling, although there are challenges to attaining a precise calibration. In a realistic situation, the PEC sphere calibration can be perturbed in various ways. Eight different scenarios including calibration COC misalignment and error in calibration ROC are described for further investigation. Perturbation scenarios are divided into 4 categories including 1—calibration ROC variation, fixed COC, 2—calibration ROC variation, fixed apex, 3—calibration transverse misalignment, and 4—calibration axial misalignment.

**Figure 3:**
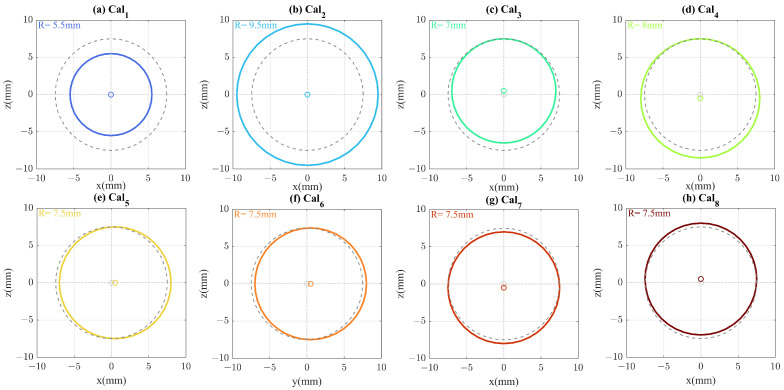
Different perturbations in the calibration sphere are shown: (**a**,**b**) calibration ROC variation, fixed COC, (**c**,**d**) calibration ROC variation, fixed apex, (**e**,**f**) calibration transverse misalignment, and (**g**,**h**) calibration axial misalignment. Correct calibration (Cal${}_{0}$) is plotted with a dashed grey line and compared with different perturbation scenarios. Its COC and ROC coincide with the cornea.

In category 1, ∓2 mm of error in calibration ROC is considered in the way that calibration sphere COC and cornea COC co-located, called Cal${}_{1}$ and Cal${}_{2}$, respectively. In category 2, an error of ∓0.5 mm in calibration ROC is considered, and calibration COC moved in the way that calibration sphere apex and cornea apex co-located, called Cal${}_{3}$ and Cal${}_{4}$, respectively. In category 3, calibration ROC is kept identical with the cornea and 0.5 mm misalignment of calibration COC in transverse location is considered, once the calibration COC is located in $x_{0}=0.5$ mm and once the calibration COC is located in $y_{0}=0.5$ mm, called Cal${}_{5}$ and Cal${}_{6}$, respectively. Finally, in category 4, the calibration COC misalignment is considered in the optical axis ($z_{0}=\mp 0.5$), called Cal${}_{7}$ and Cal${}_{8}$, respectively. Figure 3a–h demonstrates these scenarios and compares them with the accurate ROC and COC for calibration.

**Figure 4:**
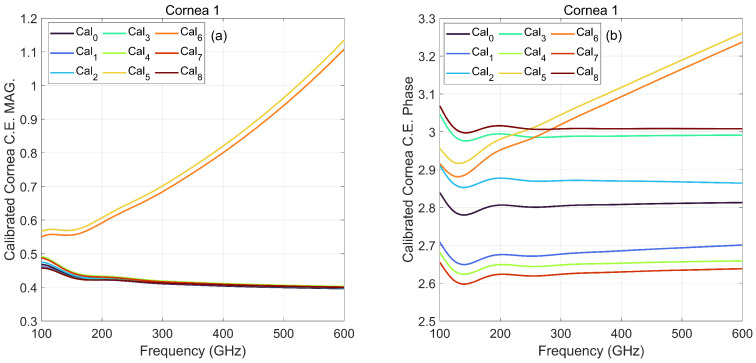
(**a**) Magnitude and (**b**) phase of cornea 1 calibrated by 8 different perturbation scenarios compared to correctly calibrated cornea, Cal${}_{0}$, at frequency range of 100–600 GHz.

In Figure 4, cornea 1 is used as a target and calibrated with various calibration scenarios at a frequency range of 100–600 GHz. A sawtooth behaviour in the phase of perturbed case 1, 2, 7, and 8 is observed which is an indicator of COC misalignment toward the optical axis or error in calibration ROC. The Cal${}_{1}$ and Cal${}_{2}$ phase is corrected by considering ∓2 mm phase shift corresponds to an error in calibration ROC. Furthermore, Cal${}_{7}$ and Cal${}_{8}$ phase is corrected by considering the ∓0.5 mm phase shift corresponds to an error in the axial location of calibration COC and is reported in Figure 4b. Phase correction revises the phase consistent with the accurate calibration phase. Interestingly, Cal${}_{3}$ and Cal${}_{4}$ did not need any phase correction because the ROC and COC change together, in a way that they cancel out the sawtooth behavior of each other and reveal a phase close to accurate calibration. The Cal${}_{5}$ and Cal${}_{6}$, transverse COC misalignment, affects the amplitude and phase stronger than the rest of the scenarios, showing an error of around 10% to more than 70% over the band with respect to the Cal${}_{0}$. Among different calibration scenarios, Cal${}_{1}$ and Cal${}_{2}$ are less sensitive to the perturbation and act closer to the Cal${}_{0}$ after phase correction.

## 4. Extraction of Corneal Features

To obtain corneal properties from the reflection spectrum, including CAWC, CPWC and CCT, the PSO algorithm is applied [14]. The Additive white Gaussian noise (AWGN) was added to the cornea and PEC sphere coupling efficiencies to build a real-life situation. The noise power was chosen to mimic scenarios in which the SNR was either 40, 50, or 60 dB. For calibration, the noisy cornea coupling efficiency was divided by the noisy PEC coupling efficiency to make it comparable to an analogous plane-wave model. This operation was repeated for six cornea targets. Then, the PSO algorithm was used to extract the target parameters by searching for the corresponding plane-wave model whose reflectivity minimizes the difference with the noisy simulated reflectivity. The adopted merit function is [22,23]:

(4) $$MF=0.33\frac{\frac{1}{N}\sum_{i=i}^{N}∥∥\Gamma_{Cal}∥-∥\Gamma_{PW}∥{∥}^{2}}{\frac{1}{N}\sum_{i=i}^{N}∥\Gamma_{Cal}{∥}^{2}}+0.66\frac{\frac{1}{N}\sum_{i=1}^{N}∥∠\Gamma_{Cal}\times e^{ikz}-∠\Gamma_{PW}{∥}^{2}}{\frac{1}{N}\sum_{i=1}^{N}∥∠\Gamma_{Cal}\times e^{ikz}{∥}^{2}},$$

where $e^{ikz}$ is added to compensate for the deviation of the calibration COC along optical axis *z* or calibration ROC deviation. $\Gamma_{Cal}$ is the noisy calibrated reflection coefficient, $\Gamma_{pw}$ is the plane-wave model reflection coefficient, and *N* is the number of frequency points. The merit function is a sum of the mean squared error normalized with the average power of the merit function metric.

### 4.1. PSO Analysis for Correctly Calibrated Cornea

**Figure 5:**
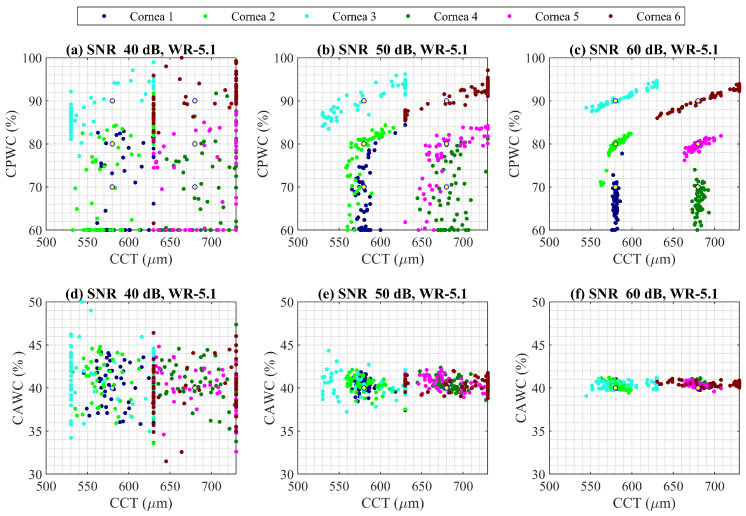
PSO analysis for extracting CPWC, CAWC, and CCT of six corneas at frequency band of WR 5.1 (140–220 GHz) considering SNR of (**a**,**d**) 40 dB, (**b**,**e**) 50 dB, and (**c**,**f**) 60 dB. Nominal values are shown with yellow dot and each cornea properties is plotted with different color.

For the six cornea properties defined in Table 2, an SNR of 40 dB, 50 dB, and 60 dB were added to target and calibration coupling efficiency and was used as an input to the PSO algorithm to extract the corneal properties. The standard frequency range of waveguide WR 5.1 (140–220 GHz) was considered. The extracted values for cornea CAWC, CPWC, and CCT are shown in Figure 5. The different corneas are plotted with different colours. Nominal values are shown with yellow spots. It is obvious that higher SNR contributes to a more accurate estimation of variables.

**Figure 6:**
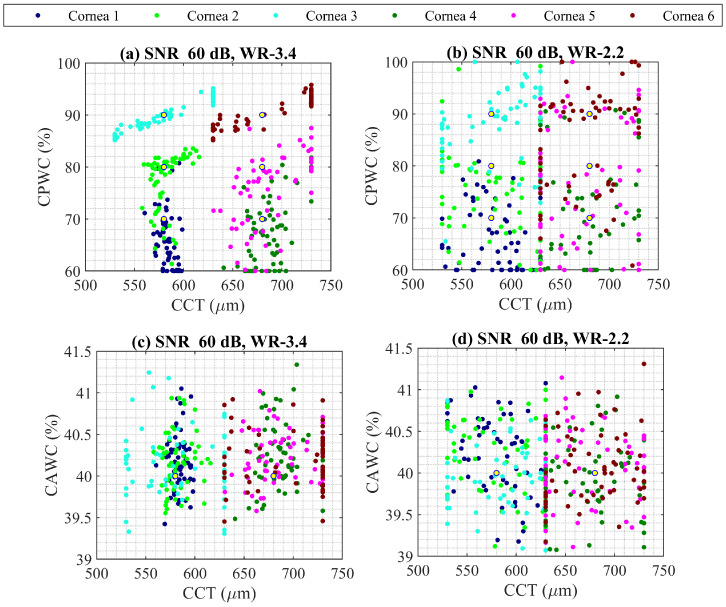
PSO analysis comparison for (**a**,**c**) WR-3.4 (220–330 GHz), and (**b**,**d**) WR-2.2 (330–500 GHz), considering SNR 60 dB for six correctly calibrated noisy corneas. Nominal values are shown with yellow dot and each cornea properties is plotted with different color.

In the following, the standard waveguide bands of WR-3.4 (220–330 GHz) and WR-2.2 (330–500 GHz) were compared for SNR of 60 dB, repeating a PSO analysis for six noisy correctly calibrated cornea, and the comparison is depicted in Figure 6. The WR-3.4 provides a better estimation of variables compared to WR-2.2, yet worse than WR-5.1.

### 4.2. PSO Analysis in Case of Perturbation

For more exploration, PSO analysis for perturbed calibrated cornea was performed. The 60 dB SNR was added to six corneas described in Table 1, then calibrated by various noisy perturbation scenarios introduced in Figure 3. Table 2, Table 3 and Table 4 reports the root-mean-square deviation (RMSD) of estimated CCT, CAWC, and CPWC from their nominal values at the frequency band of WR 5.1, WR 3.4 and WR 2.2, respectively. The consequence of the error in calibration sphere ROC and COC is elucidated in Table 2, Table 3 and Table 4. The error in parameter estimation rises significantly (around 30%), especially for transverse misalignment calibration, Cal${}_{5}$ and Cal${}_{6}$, consistent with the result in Figure 4.

## 5. Discussion

This study suggests extending the corneal spectroscopy from the previously studied WR-3.4 band to the WR-5.1 using an accurate computational method based on Fourier-optics and vector spherical harmonics and introducing an acceptable noise level of 60 dB.

Furthermore, in terahertz cornea sensing and imaging, it is suggested to calibrate the cornea reflection with a steel sphere (identical to the radius and location of the target) reflection that contributes to an enhancement in longitudinal modes resolution. The challenge to reach an optimal calibration was discussed and addressed. Perturbed scenarios affect phase significantly. Adding a phase correction coefficient (coincidence to the misalignment in optical axis or ROC calibration) contributed to the proper compensation of the phase, yet phase correction was not adequate for the transverse misalignment.

This study assists in analyzing measured reflection in the laboratory and offers some insights such as avoiding the transverse dislocation of the calibration sphere due to a higher level of the error, also mitigating the misalignment in the optical axis or the deviation of the calibration ROC through distance estimation in the PSO algorithm.

## Figures and Tables

**Table 1:** Different cornea characteristic.

Cornea	Thickness	ACWC	PCWC
Cornea 1	580 μm	40	70
Cornea 2	580 μm	40	80
Cornea 3	580 μm	40	90
Cornea 4	680 μm	40	70
Cornea 5	680 μm	40	80
Cornea 6	680 μm	40	90

**Table 2:** RMSD of estimated CCT, CAWC, and CPWC for six noisy cornea calibrated with various noisy perturbed PEC sphere at frequency band of WR 5.1. The SNR 60 dB is considered.

	Ca1${}_{1}$	Ca1${}_{2}$	Ca1${}_{3}$	Ca1${}_{4}$	Ca1${}_{5}$	Ca1${}_{6}$	Ca1${}_{7}$	Ca1${}_{8}$
CCT${}_{1}$	22.2 μm	12.7 μm	26.0 μm	24.7 μm	16.6 μm	14.9 μm	19.9 μm	22.5 μm
CAWC${}_{1}$	2.5%	6.1%	3.4%	9.7%	10.0%	10.0%	9.7%	3.8%
CPWC${}_{1}$	10.5%	9.7%	14.7%	13.4%	29.5%	29.3%	12.2%	12.7%
CCT${}_{2}$	27.3 μm	25.2 μm	32.8 μm	29.1 μm	18.7 μm	14.9 μm	28.4 μm	33.9 μm
CAWC${}_{2}$	3.0%	6.7%	2.6%	9.9%	10.0%	10.0%	9.8%	4.9%
CPWC${}_{2}$	7.2%	9.6%	8.2%	11.0%	19.3%	19.4%	10.6%	10.4%
CCT${}_{3}$	33.2 μm	39.3 μm	36.7 μm	44.0 μm	35.2 μm	32.1 μm	42.0 μm	36.1 μm
CAWC${}_{3}$	3.0%	6.7%	2.8%	9.8%	10%	10%	9.9%	3.7%
CPWC${}_{3}$	8.8%	10.3%	9.7%	6.3%	9.3%	8.9%	7.6%	11.9%
CCT${}_{4}$	15.5 μm	12.3 μm	17.4 μm	15.2 μm	49.9 μm	49.9 μm	12.2 μm	15.6 μm
CAWC${}_{4}$	1.9%	6.5%	0.9%	10.0%	10.0%	10.0%	9.9%	2.1%
CPWC${}_{4}$	8.8%	7.7%	8.3%	8.1%	29.7%	29.2%	7.7%	7.3%
CCT${}_{5}$	16.9 μm	20.0 μm	23.4 μm	22.8 μm	49.9 μm	49.7 μm	17.3 μm	22.1 μm
CAWC${}_{5}$	1.7%	6.6%	0.7%	9.8%	10.0%	10.0%	9.9%	2.0%
CPWC${}_{5}$	6.3%	9.9%	8.4%	8.9%	19.8%	19.8%	5.6%	7.5%
CCT${}_{6}$	36.9 μm	41.8 μm	40.9 μm	43.5 μm	50.0 μm	50.0 μm	42.8 μm	40.0 μm
CAWC${}_{6}$	1.44%	6.6%	0.84%	9.9%	10.0%	10.0%	10.0%	1.86%
CPWC${}_{6}$	6.2%	4.3%	7.3%	5.6%	10.3%	10.1%	4.7%	7.9%

**Table 3:** RMSD of estimated CCT, CAWC, and CPWC for six noisy cornea calibrated with various noisy perturbed PEC sphere at frequency band of WR 3.4. The SNR 60 dB is considered.

	Ca1${}_{1}$	Ca1${}_{2}$	Ca1${}_{3}$	Ca1${}_{4}$	Ca1${}_{5}$	Ca1${}_{6}$	Ca1${}_{7}$	Ca1${}_{8}$
CCT${}_{1}$	19.4 μm	23.5 μm	24.1 μm	25.2 μm	48.7 μm	48.4 μm	21.1 μm	21.6 μm
CAWC${}_{1}$	2.0 %	7.1 %	6.0 %	9.9 %	10.0 %	10.0 %	9.9 %	3.3 %
CPWC${}_{1}$	11.1 %	13.5 %	13.6 %	15.6 %	25.5 %	23.4 %	13.3 %	12.7 %
CCT${}_{2}$	30.3 μm	27.5 μm	27.3 μm	30.0 μm	48.7 μm	49.5 μm	30.2 μm	25.0 μm
CAWC${}_{2}$	2.1 %	7.4 %	6.1 %	10.0 %	10.0 %	10.0 %	9.9 %	3.3 %
CPWC${}_{2}$	12.5 %	11.3 %	13.7 %	12.2 %	16.8 %	16.8 %	12.3 %	11.9 %
CCT${}_{3}$	40.9 μm	44.3 μm	40.1 μm	47.3 μm	49.5 μm	49.4 μm	45.3 μm	39.1 μm
CAWC${}_{3}$	1.9 %	6.9 %	5.8 %	9.9 %	10.0 %	10.0 %	9.9 %	3.1 %
CPWC${}_{3}$	6.4 %	5.1 %	5.7 %	4.1 %	11.0 %	10.6 %	4.5 %	5.9 %
CCT${}_{4}$	31.1 μm	33.5 μm	32.0 μm	32.3 μm	49.3 μm	49.1 μm	32.5 μm	35.3 μm
CAWC${}_{4}$	2.1 %	7.5 %	5.9 %	10.0 %	10.0 %	10.0 %	10.0 %	3.4 %
CPWC${}_{4}$	16.1 %	17.3 %	13.3 %	12.2 %	14.3 %	14.0 %	15.2 %	15.4 %
CCT${}_{5}$	35.5 μm	37.1 μm	36.5 μm	34.7 μm	49.8 μm	49.6 μm	37.3 μm	39.0 μm
CAWC${}_{5}$	2.2 %	7.5 %	6.2 %	10.0 %	10.0 %	10.0 %	9.9 %	3.3 %
CPWC${}_{5}$	12.4 %	12.7 %	11.4 %	10.6 %	5.3 %	5.2 %	9.5 %	12.7 %
CCT${}_{6}$	44.4 μm	42.7 μm	46.0 μm	43.7 μm	49.9 μm	49.9 μm	40.2 μm	43.7 μm
CAWC${}_{6}$	1.9 %	7.4 %	6.0 %	10.0 %	10.0 %	10.0 %	9.9 %	3.3 %
CPWC${}_{6}$	10.5 %	10.8 %	9.7 %	9.9 %	6.1 %	5.5 %	9.2 %	9.3 %

**Table 4:** RMSD of estimated CCT, CAWC, and CPWC for six noisy cornea calibrated with various noisy perturbed PEC sphere at frequency band of WR 2.2. The SNR 60 dB is considered.

	Ca1${}_{1}$	Ca1${}_{2}$	Ca1${}_{3}$	Ca1${}_{4}$	Ca1${}_{5}$	Ca1${}_{6}$	Ca1${}_{7}$	Ca1${}_{8}$
CCT${}_{1}$	33.6 μm	35.7 μm	33.1 μm	32.2 μm	22.1 μm	17.4 μm	29.8 μm	34.6 μm
CAWC${}_{1}$	7.8 %	9.4 %	10.0 %	10.0 %	10.0 %	10.0 %	10.0 %	9.7 %
CPWC${}_{1}$	18.7 %	18.0 %	18.5 %	18.9 %	23.5 %	23.0 %	17.2 %	15.8 %
CCT${}_{2}$	34.4 μm	37.8 μm	36.1 μm	31.6 μm	20.7 μm	21.2 μm	35.4 μm	31.6 μm
CAWC${}_{2}$	7.7 %	9.4 %	9.9 %	10.0 %	10.0 %	10.0 %	10.0 %	9.6 %
CPWC${}_{2}$	15.9 %	14.1 %	13.7 %	13.4 %	14.1 %	14.0 %	14.3 %	15.4 %
CCT${}_{3}$	36.6 μm	39.8 μm	38.2 μm	35.7 μm	18.5 μm	16.8 μm	36.0 μm	34.9 μm
CAWC${}_{3}$	7.8 %	9.4 %	9.9 %	10.0 %	10.0 %	10.0 %	10.0 %	9.8 %
CPWC${}_{3}$	15.8 %	17.4 %	16.8 %	11.1 %	6.2 %	5.7 %	13.6 %	17.2 %
CCT${}_{4}$	36.0 μm	37.9 μm	36.2 μm	37.1 μm	38.9 μm	35.8 μm	38.2 μm	35.6 μm
CAWC${}_{4}$	7.7 %	9.3 %	10 %	10 %	10 %	10 %	10 %	9.7 %
CPWC${}_{4}$	17.3 %	18.4 %	17.6 %	20.2 %	28.4 %	29.0 %	20.7 %	16.7 %
CCT${}_{5}$	37.7 μm	34.0 μm	35.8 μm	39.5 μm	36.2 μm	34.4 μm	37.5 μm	40.8 μm
CAWC${}_{5}$	7.6 %	9.3 %	10.0 %	10.0 %	10.0 %	10.0 %	10.0 %	9.8 %
CPWC${}_{5}$	15.5 %	14.0 %	15.0 %	14.0 %	19.4 %	19.1 %	14.5 %	15.5 %
CCT${}_{6}$	35.7 μm	39.1 μm	38.1 μm	38.5 μm	36.8 μm	36.4 μm	34.3 μm	35.2 μm
CAWC${}_{6}$	7.7 %	9.3 %	9.9 %	10.0 %	10.0 %	10.0 %	10.0 %	9.7 %
CPWC${}_{6}$	17.6 %	17.1 %	15.9 %	13.1 %	9.4 %	9.6 %	14.6 %	16.9 %

## Data Availability

Not applicable.

## Abbreviations

The following abbreviations are used in this manuscript:

AWGN	Additive White Gaussian Noise
CAWC	Corneal Anterior Water Content
CCT	Corneal Central Thickness
COC	Center of Curvature
CPWC	Corneal Posterior Water Content
PSO	Particle Swarm Optimization
RMSD	Root-Mean-Square Deviation
ROC	Radius of Curvature
SNR	Signal to Noise Ratio
